# Surveillance of severe maternal morbidity and maternal mortality in maternity hospitals of the Latin American and Caribbean network - Red CLAP: study protocol

**DOI:** 10.1080/16549716.2023.2249771

**Published:** 2023-09-18

**Authors:** Suzanne J. Serruya, Bremen de Mucio, Claudio Sosa, Mercedes Colomar, Pablo Duran, Rodolfo Gomez Ponce de Leon, Alicia Aleman, Adriana G. Luz, Renato T. Souza, Maria L. Costa, José G. Cecatti

**Affiliations:** a CLAP/WR - Latin American Center for Perinatology, Women’s and Reproductive Health of the Pan American Health Organization (PAHO), Montevideo, Uruguay; b Department of Preventive and Social Medicine, School of Medicine, Universidad de la Republica, Montevideo, Uruguay; c Department of Obstetrics and Gynecology, University of Campinas Medical School, Campinas, Brazil

**Keywords:** Severe maternal morbidity, potentially life-threatening conditions, maternal near-miss, maternal mortality, Latin america

## Abstract

The sustained reduction in maternal mortality in America underlines the need to analyse women who survived a complication that could have been fatal if appropriate and timely care had not been taken. Analysis of maternal near-miss (MNM) cases, as well as potentially life-threatening conditions (PLTC), are considered indicators for monitoring the quality of maternal care. The specific objective of this study protocol is to develop a surveillance system for PLTC, MNM and maternal mortality, as primary outcomes, in Latin American and Caribbean maternal healthcare institutions. Secondarily, the study was designed to identify factors associated with these conditions and estimate how often key evidence-based interventions were used for managing severe maternal morbidity. This is a multicenter cross-sectional study with prospective data collection. The target population consists of all women admitted to health centres participating in the network during pregnancy, childbirth, or the postpartum period. Variables describing the sequence of events that may result in a PLTC, MNM or maternal death are recorded. Relevant quality control is carried out to ensure the quality of the database and confidentiality. Centres with approximately 2,500 annual deliveries will be included to achieve a sufficient number of cases for calculation of indicators. The frequency of outcome measures for PLTC, MNM and maternal mortality and their confidence intervals and differences between groups will be calculated using the most appropriate statistical tests. Similar procedures will be performed with variables describing the use of evidence-based practices. Networking creates additional possibilities for global information management and interaction between different research groups. Lessons can be learned and shared, generating scientific knowledge to address relevant health problems throughout the region with provision of efficient data management.

## Introduction

Between 1990 and 2015, the Millennium Development Goal (MDG) 5 aimed at reducing the maternal mortality ratio (MMR) by 75% (MMR) across the world. The MDG era ended in 2015, a propitious time to reflect on the progress achieved. The World Health Organization (WHO) estimated that more than 300,000 maternal deaths globally [1]. Around ten million women suffered from complications related to pregnancy, childbirth and postpartum worldwide [1,2].

A sustained reduction in maternal mortality in the world, and especially in America, raises the need to analyse cases of women who survived a complication that could have been fatal if proper and timely care had not been taken [1,3]. In the Americas, there is a link between death and severe maternal morbidity, a phenomenon that may be due to insufficient quality of obstetric care [4–6]. Analysis of maternal near-miss (MNM) cases, as well as potentially life-threatening conditions (PLTC), are considered indispensable indicators for monitoring the quality of maternal care processes [4–6].

In 2011, the PAHO unanimously approved a regional action plan to accelerate the reduction in maternal mortality (MM) and severe maternal morbidity (SMM). This plan was designed by the Latin American Center for Perinatology (CLAP)/Women’s and Reproductive Health (WRH) with input from PAHO country offices, technicians from women’s health programmes, maternal health staff from ministries of health, the WHO, other UN agencies, and bilateral agencies. CLAP/PAHO has defined a set of indicators to measure the progress and impact of Regional Plan implementation, allowing a comparison of information beyond local adaptation [7]. Progress reports have shown that most countries do not have routine data on severe maternal morbidity at a national level. When reports were made, notification problems existed. Very low numbers were notified with data heterogeneity in registration forms [8].

As already recognised, the need for an early diagnosis/identification of a near-miss condition has led to the definition of a potentially life-threatening condition (PLTC) that can progress to a MNM event and eventually maternal death [9]. For this reason, healthcare during pregnancy and childbirth remains essential for assurance of a normal and healthy development. Furthermore, it is important for the prevention, prediction or diagnosis of possible complications during pregnancy, childbirth or the postpartum period.

Most of these conditions are controlled by preventing the development of a more severe disorder. Well-known interventions, supported by high-quality evidence, are employed [10]. The frequency with which these conditions progress to a MNM event or death is a valid indicator to evaluate and improve the quality of healthcare services [9].

The purpose of CLAP/WRH is to promote, strengthen and/or increase the healthcare capacity of countries in the Region of the Americas. It focuses on the family, women, mothers along with their newborns. From an efficiency and equity viewpoint, it also involves men [11]. As a United Nations unit, the role of CLAP/PAHO is to provide health recommendations to countries in the region. This paper proposes a regional work platform focused on building a network of institutions and healthcare centres to improve maternal and neonatal health. Three components are developed monitoring of maternal and neonatal care, training in the generation and use of evidence-based interventions, and research.

## Rationale

Within this framework, the Red CLAP was created. It is a network of sentinel centres for women and newborns in Latin America and the Caribbean. Networking creates additional possibilities for global information management. This includes an interaction between different groups aimed at sharing lessons learned and generating scientific knowledge. Relevant regional health problems can thus be addressed by efficient data management.

Estimates of the occurrence of PLTC and MNM, their progression and maternal mortality in a continuum of severity, in addition to the frequency of use of evidence-based interventions, are useful indicators for monitoring maternal health and quality of maternal healthcare. These health events are identified in healthcare services and registered in the SIP, and more recently modified by CLAP to include these variables.

## Objectives

Therefore, the main purpose of this study is to develop a continuous monitoring system for severe maternal morbidity and mortality, including both potentially life-threatening conditions and MNM events. The primary outcome of the study was maternal mortality. It is useful for monitoring and improving quality of care and health outcomes in maternal healthcare institutions in Latin America and the Caribbean. Additionally, the study was designed to secondarily identify factors associated with these conditions, estimating the frequency of use of some key evidence-based interventions for management of severe maternal morbidity conditions. All this information will feed an electronic central database, in which periodical reviews and analysis are conducted for scientific orprogramme evaluation.

## Methods/design

The surveillance of SMM and maternal mortality in Red CLAP hospitals is a multicenter cross-sectional study with prospective data collection. Institutions participating in the research must be third-level care centres. Red CLAP members who sign a participation agreement are committing themselves to carry out activities established in the protocol and operations manual. In addition, institutions must have at least 2500 annual deliveries. Second-level care institutions that retrieve data from women who meet inclusion criteria and require transference to high-complexity centers may also participate. All centres that use the most recent version of the data collection form from the Perinatal Information System (SIP – containing information for PLTC and MNM identification) or a similar system may participate as a clinical registry. Centres that do not use this system but are committed to collecting the same variables used by SIP for the Module of Morbidity and Interventions may also participate. The main primary outcome variables are SMM (PLTC, MNM and MM). The secondary outcome is the use of defined therapeutic or preventive evidence-based practices (magnesium sulphate for severe preeclampsia or eclampsia, antibiotics for sepsis, uterotonic agents after birth and for postpartum haemorrhage, etc.). Other sociodemographic, epidemiological, and clinical variables of interest may also be included and assessed as potential predictors for severe maternal morbidity and its different components. Variables that describe the sequence of events or conditions leading to MNM or maternal death [9] will be measured (Supplementary Material – Appendix 1).

### Procedures for data collection

All study data will be collected in the SIP or a compatible system considered reliable by the institution. All women who enter the service due to the threat of abortion, ectopic pregnancy, trophoblastic disease, complications of pregnancy (regardless of gestational age), labour, or postpartum period until discharge (group of interest) or without any complications (group for comparison) will routinely initiate a perinatal clinical history that will be completed during the healthcare process using the SIP. For a proposed analysis similar to the existing analytical approach, it is fundamental to generate incidence rates for all types of morbidity. Furthermore, the reference group generates SMM risk ratios, according to predictive variables. Upon completion of registration, data is entered into electronic records to build a database. All registered cases will constitute a study database. In case the institution has an electronic history, this process will preferably occur in real-time.

The Perinatal Information System (SIP) is a free PAHO product developed by the Latin American Center of Perinatology (CLAP/SMR) to record perinatal care from the first antenatal visit to patient discharge, contributing to timely decision-making. When a pregnant woman is initially checked, the medical record is simultaneously entered into a computer (or any other mobile device). Medical records can then be transmitted online or deferred with maximum security and privacy guarantees [12].

Data management is carried out according to the situation of each institution. In institutions with a good and constant connectivity, that are capable of incorporating a Web system, real-time data are sent automatically to CLAP, respecting confidentiality aspects. When connectivity is intermittent, data can be sent automatically to CLAP, without even using the SIP Web. Once the base is backed up, sent to CLAP and entered into the Data Coordinating Center (DCC) server, the system generates an identification number. Thus, the registers are automatically anonymised for use in the CLAP data unit. With this identification number, national centres can restore the record identity of cases that should be corrected or completed.

The system will identify cases of data duplication when consolidating the records submitted. In case of more than one record with the same identification number, it will save the newest case. The computer system will be configured to make the study mandatory. If variables were not recorded, the form cannot be definitively saved. Theprogramme generates alarms to show other inaccuracies or impossible or unlikely answers (such as maternal age = 8 or 65), or even other logical inconsistencies (a woman who died in the hospital could not have been discharged with contraceptive guidance).

To ensure a complete and high-quality database, the DCC will perform a data recovery process that follows the Good Clinical Practice guidelines in research. Completeness/correction of lacking data or inconsistencies will be carried out locally in participating institutions and directly in the database. Data are sent weekly with a report of inconsistencies, which are then corrected.

Training for each centre coordinator involved in filling out the instrument is offered. Data collectors are trained by the coordinators. An operation manual is provided. This includes a general study description, a step-by-step description of the accurate and complete form-filling process and filling controls to be performed immediately after patient discharge. The form will be the same as that already used routinely in the SIP, with the addition of sessions on PLTC and MNM, as previously described.

### Data management and sample quality control

General data management is carried out by the DCC located in the CLAP. The centre will be in charge of generating, organising, controlling, consolidating, guarding and maintaining the database. It will also maintain database quality, ensuring subject confidentiality. It will also be responsible for sending any inconsistencies detected for correction and/or completion. The institution will periodically receive a list of discrepancies from records received by the DCC. Requested data is retrieved and the corrected database is sent back to the DCC. When new data is consolidated, records with omissions are replaced by correct data.

For quality control, CLAP will randomly and periodically ask some Institution coordinators to send pictures of the birth logbook from selected days. The reason is to check whether deliveries occurring on that day actually coincide with database records. Additionally, an external evaluator will periodically perform an audit visit to the institution for review of clinical record samples, assessing whether these are in agreement with the records sent. Quality control will be completed with corrections of inconsistencies.

### Study organization

**Figure 1.**
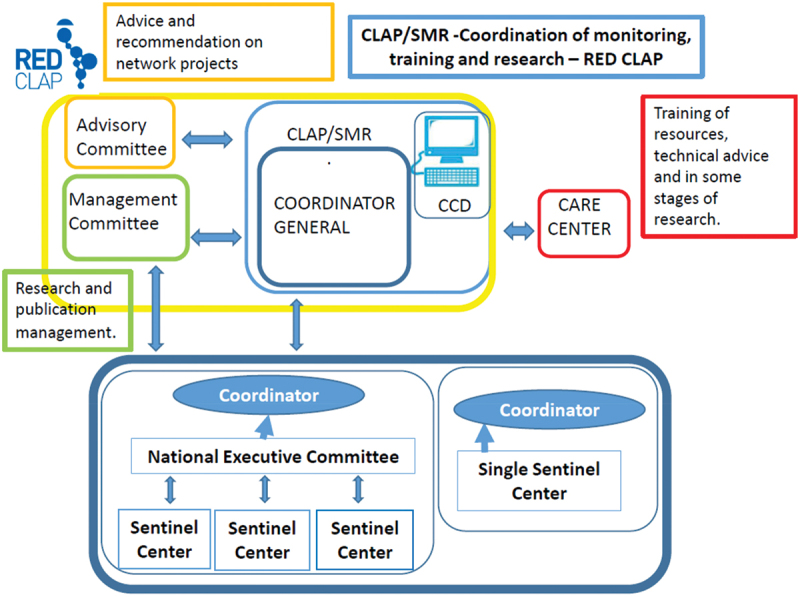
Study organization.

Each institution should have a local coordinator. This individual will be in charge of coordinating the whole data collection process, including data submission, ensuring the quality of filling out data and controlling compliance with the protocol and possible deviations. The coordinator will maintain communication with the DCC and will be in charge of dialogue with the national executive committee and the Steering Committee. The general coordination will be carried out by the Red CLAP managing committee. It will be in charge of following the study globally and discussing data. In addition, it will participate in the final report writing and coordinate publications that arise from this study. It will maintain a direct dialogue with the National Executive Committee and Coordinator (Figure 1).

A steering meeting will be held before the new system is used and data are collected. A trial period of the system will occur in selected centres with a new virtual meeting at the time of initiation. Virtual monitoring meetings will be held regularly. Likewise, in a face-to-face meeting with coordinators of the local teams, implementation of the research protocol will be discussed and the staff will be trained in instrument use, filling out and analysing data in the Perinatal Computer System.

## Data analysis

### Sample size

It is estimated that the minimum sample size should allow the attainment of at least 20 events of severe maternal outcomes (SMO: near miss cases plus maternal deaths) at each centre during the data collection period. Considering that the estimated prevalence of severe maternal outcomes (SMO) is 7.5 cases per 1000 births, when 2000 women are screened, we expect to identify 15 SMO (95% CI 7–30). Therefore, we estimate that it would be necessary to include centres with at least 2500 annual deliveries to obtain the required number of events [13]. With these parameters, data collection over 1 year will provide enough information to estimate the occurrence of SMM in most participating centres. Despite the relatively small number of outcome events (SMO), in theory, each hospital would have enough data to generate indicators of the proposed outcomes. A specific number of participating hospitals are thus not necessary.

### General analysis strategy

Following primary outcome assessment, women classified as SMM and its different subgroups (PLTC, MNM or MD) will be the group of interest in this project, whereas all other women will be the control or reference group. The control group has no serious complications; therefore, it will serve as a comparison in all analyses. Institutional descriptive statistics are estimated to describe sociodemographic profile of the women. Percentages and means are estimated for categorical and continuous data with confidence intervals and standard deviations, respectively. The frequencies of PLTC, MNM, SMO and MM, with their confidence intervals (95% CI) and differences between groups of women with and without these conditions will be calculated. We use different statistical tests to evaluate the significance of differences between groups (t-test and ANOVA for continuous data of two or more levels and chi-square or ANOVA for categorical variables of 2 or more levels). Similar procedures will be performed with variables describing evidence-based practices. The relationship between the use of these interventions and maternal and perinatal outcomes will be evaluated by estimation of prevalence ratios (PR) (crude and adjusted), with their 95% confidence intervals. Multivariate analyses are performed to identify the association between variables with PLTC, MNM and MM. The positive and negative likelihood ratios of SMM for the new set of PLTC variables will be re-calculated. Inter-cluster correlation coefficient values will be taken into account, since data will be coming from multiple centres, some from the same country using Generalised Estimation Equation (GEE) models.

Indicators of severe maternal conditions will be automatically calculated by the system:

- the MMR/100.000 live births (number of MD/100,000 LB)
- MNM ratio (number of MNM/1000 LB),
- Severe Maternal Outcomes ratio (near-miss + maternal deaths/1000 LB)
- MNM to MD ratio (Number of MNM per MD occurred)
- Mortality rate (number of maternal deaths/numbers of near-miss + maternal deaths per 100)
- criterion/case ratio (total inclusion criteria for MNM/total cases with MNM),
- PLTC ratio (number of PLTC/1000 LB)

## Ethical aspects

Data will be obtained from medical records used in the medical care process in an observational study. There will be no personal identifiers of the study participants. Data collectors will fill out SIP records with medical history data also used in women’s healthcare. Personnel may be required to retrieve unregistered data and review clinical records. Professionals participating in the study will be asked to sign a conflict of interest declaration, detailing and maintaining confidentiality of the data collected. Institutions will only participate if the protocol is approved by the local Ethics Committees. The present protocol was submitted and approved by PAHO Ethics Review Committee in August 2018 (PAHOERC Ref. No: PAHO-2018-04-0025). It also waived the need for an informed consent form for each participating woman, considering that data will be collected after hospital discharge. Information will probably represent an important social value for dealing with maternal morbidity and mortality, and the study does not represent any risks to participants.

## Funding

The SIP resource is already implemented and functioning in its most recent version under CLAP/PAHO monitoring, providing free access to all Latin American and Caribbean countries. It was mainly designed to improve the quality of care in the region for women and children during pregnancy and postpartum. Study development involving Red CLAP was then financially supported by CLAP/PAHO itself without a specific research grant. Centres were voluntarily affiliated and did not receive any additional financial support, apart from team training and access to the system. Each hospital/institution that wishes to participate needs to follow the previously described requirements. It requires basic access to computer and internet resources. The appointment of basic staff responsible for feeding the system with information and data management is needed. Specifically, planned analyses should have responsible researchers in search of additional resources for implementation.

## Dissemination of results

A strategy for disseminating results will be elaborated, focusing on different audiences (governments, scientific communities, health professionals, general public) and diverse means and types of communications. In addition, a final meeting is planned for RedCLAP participating professionals when the results will be initially shown. These results will also be presented at national and regional conferences and meetings. Scientific articles will be produced for publication in open-access journals concerning the main and secondary objectives of the study. If possible, results will also be available on national and international websites for discussion with policymakers and professionals from included institutions. There has already been agreement on authorship rules, based on established Red CLAP criteria, and in compliance with journal requirements.

## Discussion

To improve maternal and perinatal health, access to a minimum set of timely, high – quality data is required to inform policy and programme decisions. Routine data collection from health systems in most low – and middle – income countries is characterised by poor data quality and completeness. Furthermore, it does not include important variables for the assessment of relevant health conditions. These are the reasons why routine data collection has not been recommended in many settings to track key outcomes and coverage indicators. It is not considered a valuable source for surveillance and research.

However, when limitations of these databases are addressed, ensuring high-quality data and coverage, routine use of data collection will surpass the needs of a health system. Routine health data collection has potential advantages, including availability at a relatively low cost and continuity at the facility level. Furthermore, when data is collected in digital registries, taking into consideration health systems, surveillance and research needs, it is possible to have real-time analysis, timely identification of alerts and monitoring of health outcomes in a permanent and accurate manner. Red CLAP deals with limitations of routine data collection assuring data quality and providing continuous monitoring, analysis and information to the network environment.

The Millennium Development Goals (MDGs) have been a powerful stimulus for American countries. Health surveillance records and procedures for women and their children have been optimised, contributing to a greater and better identification of factors that determine maternal death. More reflection has been directed, raising awareness of factors involved in SMM.

Several scientific investigations have produced insights into risk factors and causes of MM and SMM that transcend women’s biological conditions. In conclusion, prevention largely depends on the response capacity of health units. A larger number of studies on the quality of services and response capacity of health professionals are required.

On the other hand, the post-2015 MDG agenda includes the implementation of the Sustainable Development Goals (SDG) agenda and sets more ambitious targets for the period 2015–2030. Additional priorities have been established, including enhancing the quality of sexual and reproductive health and rights, reducing the prevalence of communicable diseases and their risk factors, and improving mental health.

The opportunity to contribute to policy formulation to decrease MM and SMM in the region has encouraged CLAP to submit a proposal for technical cooperation with other countries. The goal is to operate an epidemiological surveillance network, based on the development of health professional skills. Furthermore, the aim is to analyse and systematise evidence, good practices and capacity building for applied research development. Highly effective decisions and public policy management are supported to create a knowledge platform for the benefit of PAHO/WHO regional technical cooperation policy.

Networking creates additional possibilities for global information management, as well as interaction between different research groups that share lessons learned and generate scientific knowledge. Increased knowledge will address the relevant health problems throughout the region by providing efficient data management [14]. The network provides a very good framework for collaborative work between countries that have already been implemented and is using the information system for years. Latin American countries have a history of joint work in health stimulated by PAHO, meaning that previous experience already exists. This situation facilitates network activities.

The main limitation of this network is related to data representativeness. Not every country is expected to have data with representation at a national level. However, surveillance systems based on sentinel centres will enable the detection of alarms, contributing to an early detection and quick response to acute situations.

## Supplementary Material

Supplemental Material

## Paper context

Current knowledge of maternal death alone is no longer sufficient to provide maternal health assessment. It is necessary to monitor the occurrence of several degrees of maternal complications that could lead to death if no appropriate and timely intervention is provided. This protocol describes a network created for surveillance of these complications in Latin American and Caribbean countries, to make changes in policies and improve maternal and perinatal health.
