# Supplementary material for: Surveillance of severe maternal morbidity and maternal mortality in maternity hospitals of the Latin American and Caribbean network - Red CLAP: study protocol
Source: Glob Health Action. 2023 Sep 18;16(1):2249771. doi: 10.1080/16549716.2023.2249771 (PMC10512792; doi:10.1080/16549716.2023.2249771)
Supplement: Supplemental Material [file ZGHA_A_2249771_SM3833.docx]

**Appendix S1.** The WHO criteria for potentially life-threatening conditions and maternal near miss [9]

1. **Criteria for potentially life-threatening conditions**

**Hemorrhagic disorders**

Abruptio placentae

Accreta/increta/percreta placenta

Ectopic pregnancy

Postpartum Hemorrhage

Ruptured uterus

**Hypertensive disorders**

Severe preeclampsia

Eclampsia

HELLP syndrome

**Other systemic disorders**

Endometritis

Seizures

**Cont. Other Systemic disorders**

Sepsis

Shock

Thrombocytopenia <50.000

Thyroid crisis

**Severe Management Indicators**

Blood transfusion

Central venous access

Hysterectomy

ICU admission

Prolonged hospital stay (>7 postpartum days)

Non-anesthetic Intubation

Surgical intervention

1. **Criteria for maternal near miss criteria**

**Clinical criteria**

Acute cyanosis

Gasping

Respiratory rate >40 or <6/min

Shock

Oliguria non responsive to fluids or diuretics

Clotting failure

Loss of consciousness lasting ≥12 hours

Loss of consciousness AND absence of pulse/heart beat

Stroke

Uncontrollable fit/total paralysis

Jaundice in the presence of pre-eclampsia

**Laboratory-based criteria**

Oxygen saturation <90% for ≥60 minutes

pH <7.1

**Cont. Laboratory-based criteria**

PaO2/FiO2 <200 mmHg

Lactate >5

Creatinine ≥300 mmol/l or ≥3.5 mg/dl

Acute thrombocytopenia (<50 000 platelets)

Bilirubin >100 mmol/l or >6.0 mg/dl

Loss of consciousness AND the presence of glucose and ketoacidosis in urine

**Management-based criteria**

Use of continuous vasoactive drugs

Hysterectomy following infection/hemorrhage

Transfusion of ≥5 units red cell transfusion

Intubation and ventilation for ≥60 minutes not related to anesthesia

Dialysis for acute renal failure

Cardio-pulmonary resuscitation (CPR)
